# Integrative Machine Learning Framework Revealing TRPM4-Associated Signatures and Identifying SPATA6 as a Potential Biomarker in Prostate Cancer

**DOI:** 10.7150/jca.129356

**Published:** 2026-05-11

**Authors:** Hang Zhou, Wangli Mei, Jichen Wang, Xiang Liu

**Affiliations:** 1 Department of Urology, Putuo People's Hospital, School of Medicine, Tongji University, Shanghai, 200061, China.; 2 Department of Urology, Shanghai East Hospital, School of Medicine, Tongji University, Shanghai, 200120, China.; 3 Senior Department of Urology, the Third Medical Center of PLA General Hospital, Beijing, 100039, China.

**Keywords:** Prostate cancer, TRPM4-realted signatures model, sodium overload, Machine Learning, SPATA6

## Abstract

**Background:**

The non-selective cation channel TRPM4 can induce necrotic cell death through sodium overload, yet its role in prostate cancer (PCa) progression remains poorly characterized.

**Materials and methods:**

Using TCGA-PCa transcriptomic data centered on TRPM4, we identified transcriptional signatures linked to sodium overload. Leveraging prognostic features, we developed robust prognostic models via ten machine learning algorithms and their combinations, training on TCGA data and validating on internal validation set, GSE46602 and GSE116918. We assessed the model's associations with clinicopathological features, prognosis, immune infiltration, and drug response. Expression of the 10 key model genes was validated in PCa cell lines versus a normal prostate epithelial cell. For SPATA6—the top-contributing gene—we overexpressed it in PCa cells to assess its functional impact.

**Results:**

We identified 91 overlapping genes from TRPM4-associated and PCa-related differentially expressed genes. Functional enrichment implicated these genes in small GTPase activity, Rap1 signaling, and cAMP signaling. A TRPM4-related signature model (TRSM) comprising 10 key genes demonstrated strong prognostic performance across training and validation cohorts. TRSM-based risk stratification revealed significant differences in disease-free survival, clinicopathological features, immune infiltration, and immunotherapy response. Drug sensitivity analysis indicated heightened docetaxel sensitivity in the high-risk group. *In vitro* assays confirmed downregulation of all 10 key genes in PCa. SPATA6 overexpression suppressed PCa cell proliferation and migration.

**Conclusion:**

Our findings underscore the importance of TRPM4-associated molecular features in PCa prognosis. TRSM shows potential as a predictive tool for patient outcomes and a guide for personalized therapy.

## Introduction

Among the most common malignancies diagnosed, prostate cancer (PCa) is a major cause of cancer-related mortality for men globally 1. Although early detection and treatments—including radical prostatectomy and androgen deprivation therapy—have improved outcomes for localized disease, advanced or metastatic PCa continues to present major therapeutic challenges 2, 3. Many patients eventually develop castration-resistant PCa (CRPC), an aggressive and lethal stage with limited effective treatment options and poor survival 4. While localized PCa is often associated with favorable outcomes, advanced disease is marked by therapy resistance and a dismal prognosis 5. This pronounced clinical heterogeneity underscores the limitations of existing staging systems and highlights the urgent need for more precise molecular biomarkers to predict disease aggressiveness. Such tools are essential to guide personalized therapy and improve survival in high-risk PCa patients.

In recent years, cell death mechanisms have continued to be a major focus in cancer research. Beyond the classical forms of necrosis and apoptosis, novel regulated cell death modalities—including ferroptosis, cuproptosis, and disulfidptosis—have been progressively identified and characterized 6-8. A research team in China, starting from the small-molecule necroptosis inducer necrocide 1, proposed a novel form of regulated necrosis termed “necrosis by sodium overload” (NECSO) and identified TRPM4 as a key mediator in this process 9. TRPM4, a non-selective cation channel located on the plasma membrane, has since been implicated in cell death across multiple cellular contexts 10-12. As early as 2019, Gao et al. 13 highlighted the emerging role of TRPM4 in cancer. While they observed that TRPM4 upregulation enhances sodium influx and causes membrane depolarization, the authors suggested that its primary oncogenic effects are mediated through disruption of intracellular calcium homeostasis. Subsequent studies have shown that TRPM4 contributes to PCa progression by facilitating the transition from prostatic intraepithelial neoplasia to invasive carcinoma 14-18. Although more recent work has not extensively explored sodium dynamics, it further underscores the critical involvement of TRPM4 in PCa cell death 19.

**Figure 1:**
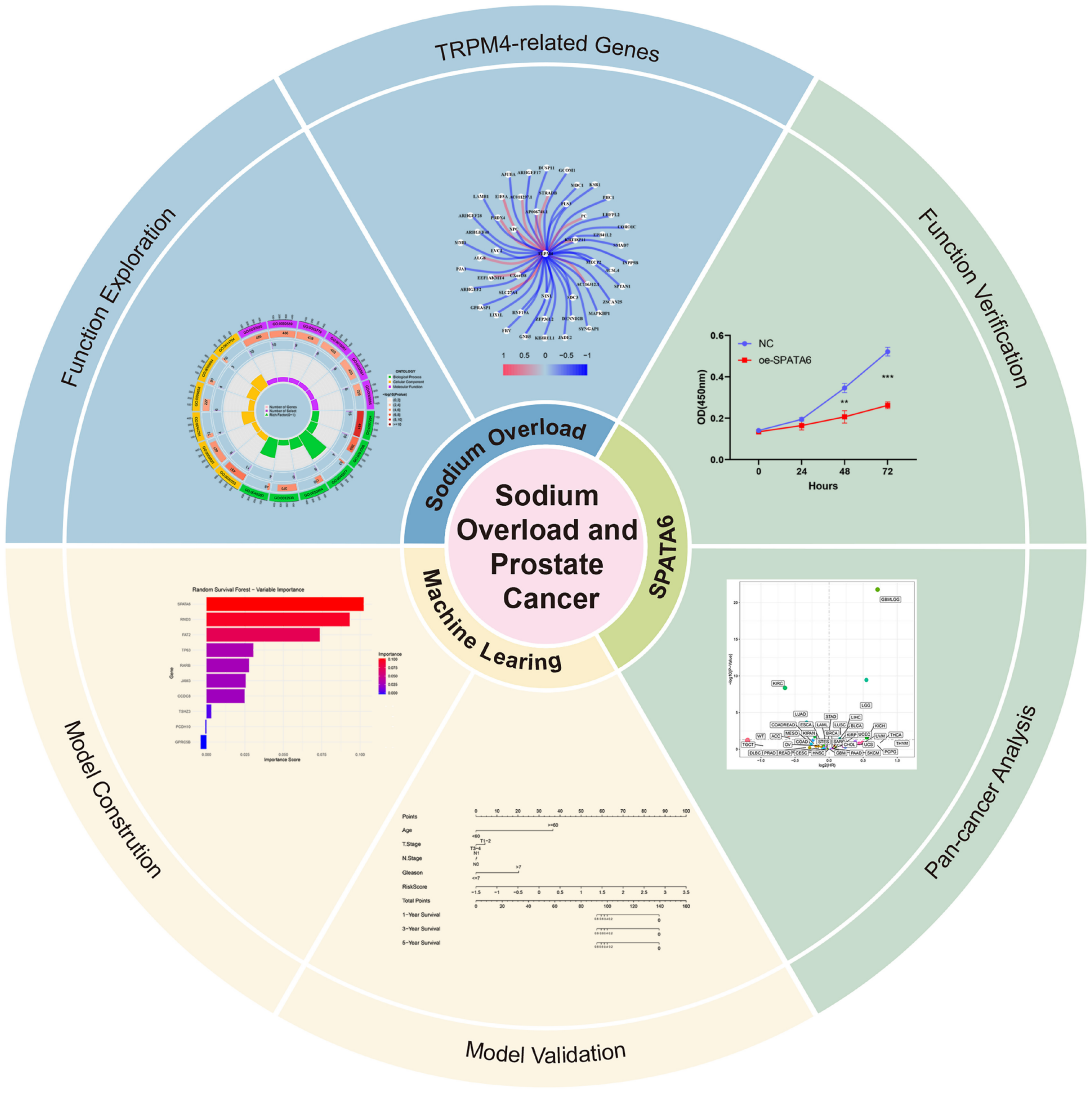
The overview of this research.

In this study, we leveraged TRPM4 as an anchor to identify TRPM4-associated molecular features from PCa transcriptomic data. Using prognostic screening and machine learning approaches, we developed a TRPM4-related signatures model (TRSM). It is worth noting that multi-omics integration has already played a significant role in tumor research 20, 21. Patients were stratified according to TRSM risk scores, and we further examined the association between risk subgroups and clinicopathological features as well as immune infiltration landscapes. A schematic of the overall study design was presented in **Figure 1**.

## Materials and Methods

### Collection of single-cell and transcriptomic data

Single-cell RNA sequencing data from PCa tissues were sourced from the GSE176031 dataset and processed using the “Seurat” R package. Key transcriptional features were extracted through principal component analysis, followed by batch effect correction with the “harmony” package. Cell clusters were visualized after dimensionality reduction using the UMAP algorithm.

Transcriptomic profiles and single nucleotide variant (SNV) data for PCa patients were obtained from The Cancer Genome Atlas (TCGA). Genes associated with TRPM4 were identified from the TCGA-PRAD cohort using a correlation threshold of |r| > 0.3 and p < 0.001. For external validation of the prognostic model, two independent PCa cohorts (GSE116918 and GSE46602) were incorporated. Additionally, immunotherapy response data were retrieved from the IMvigor210 cohort.

### Enrichment analysis of key genes

To characterize the functional profiles of TRPM4 transcriptionally correlated genes, we performed Kyoto Encyclopedia of Genes and Genomes (KEGG) and Gene Ontology (GO) enrichment analyses using the “clusterProfiler” R package. Pathway activities were further investigated through gene set enrichment analysis (GSEA) and single-sample GSEA (ssGSEA). In addition, Gene Set Variation Analysis (GSVA)—an unsupervised method for pathway-level quantification—was applied to evaluate pathway activity on a per-sample basis.

### Construction and validation of the TRSM

Using univariate Cox regression, we identified ten genes with potential prognostic value. The TCGA-PRAD cohort was designated as the training set, with GSE116918 and GSE46602 serving as independent validation cohorts. In addition, to more rigorously evaluate the stability and generalization capability of the model, 30% of the samples from the TCGA training dataset were randomly selected to form an internal validation set. To enhance predictive robustness, we integrated ten machine learning algorithms to generate multiple prognostic feature sets. Models were evaluated and ranked according to the mean concordance index (C-index) across both training and validation cohorts. In light of the relatively small feature space and sample size, the RSF algorithm was ultimately selected for model building, a decision based primarily on its cross-validation performance rather than fit to the training set. The final model—termed the TRPM4-related signature model (TRSM)—was built using the RSF algorithm and comprises ten genes: SPATA6, RND3, FAT2, TP63, RARB, JAM3, CCDC8, TSHZ3, PCDH10, and GPRC5B.

The RSF model was implemented using the “rfsrc” function from the “randomForestSRC” package in R (version 4.3.3). Several hyperparameters were specified. The number of trees was set to 900 to ensure ensemble stability and convergence of variable importance measures, consistent with the recommendations in the randomForestSRC documentation. The minimum node size was set to 15; this parameter controls the depth of individual trees and was selected to balance model complexity with generalizability, as a larger node size yields simpler trees that reduce the risk of overfitting while maintaining predictive performance. The splitting rule was set to "logrank", which is specifically designed for survival outcomes by maximizing the log-rank statistic between daughter nodes, making it the standard choice for survival random forests.

To assess clinical applicability, patients in each cohort were stratified into high-risk and low-risk groups based on median TRSM scores. Kaplan-Meier survival analysis was performed using the "survminer" R package. Spearman correlation analysis was employed to evaluate associations between TRSM scores and clinical variables, including age, T stage, N stage, M stage, and Gleason score. In the TCGA-PRAD cohort, both univariate and multivariate Cox regression analyses established TRSM as an independent prognostic factor for PCa. Finally, we constructed a clinically translatable nomogram that integrates TRSM with key clinical parameters (age, T stage, N stage, and Gleason score) to facilitate individualized prognosis prediction.

### Analysis of Genomic Variation

To quantify intratumoral genetic heterogeneity, we calculated the Mutation Allele Tumor Heterogeneity (MATH) score using whole-exome sequencing data, which reflects the distribution width of somatic variant allele frequencies. We then applied the "Maftools" R package to characterize and visualize the distinct mutation profiles between high-risk and low-risk PCa groups as defined by TRSM stratification.

### Mutated and immune infiltration landscape of different risk groups

We applied the ESTIMATE algorithm to compute immune and stromal scores, along with tumor purity, in PCa patients. Immune cell infiltration levels were assessed using ssGSEA and CIBERSORT. Furthermore, by integrating single-cell RNA sequencing data, we examined the expression patterns of the 10 signature genes across distinct cell populations within the tumor microenvironment.

### Immunotherapy response and drug sensitivity analysis

To evaluate the clinical utility of TRSM in predicting immunotherapy response, we analyzed data from the IMvigor210 cohort. Additionally, using the "pRRophetic" R package, we estimated half-maximal inhibitory concentrations (IC₅₀) for commonly used agents—including cisplatin, docetaxel, and metformin—to compare drug sensitivity between TRSM-defined risk subgroups.

### Pan-cancer analysis of SPATA6

Using transcript-per-million (TPM) data from tumor and matched normal tissues across 29 cancer types in TCGA, we analyzed SPATA6 expression patterns. We further examined the association between SPATA6 expression levels and disease-free survival (DFS) using available clinical outcome data.

### Cell lines and cell culture

Human PCa cell lines PC3, LNCap, and 22RV1 were provided by the Urology Laboratory of Shanghai East Hospital. All cells were maintained in RPMI 1640 medium supplemented with 10% fetal bovine serum at 37°C in a humidified 5% CO₂ atmosphere.

### Cell transfection

SPATA6 was cloned into the PGMLV-CMV-MCS-PGK-Puro lentiviral vector, with the empty vector used as a control. Lentiviruses were packaged in HEK293T cells, followed by transduction of PC3 and 22Rv1 cells. Stable polyclonal populations were selected using 2 μg/mL puromycin for two weeks, and transfection efficiency was confirmed by Western blot analysis.

### Western blotting

Total protein was extracted from cells using RIPA lysis buffer, separated by 10% SDS-PAGE, and transferred to a PVDF membrane. After blocking with 5% non-fat milk, membranes were probed with the following primary antibodies: beta-actin (Proteintech) and SPATA6 (Proteintech).

### Quantitative real-time polymerase chain reaction (qRT-PCR)

Total RNA was isolated using a commercial RNA extraction kit, and cDNA was synthesized with a reverse transcription kit. Quantitative polymerase chain reaction (qPCR) was performed, and expression data were normalized to an internal control and analyzed via the 2-ΔΔCT method. All primer sequences are listed in **Table S1**.

### CCK-8 assay

Cells from experimental and control groups were seeded at 2,000 cells per well in transparent 96-well plates. Absorbance at 450 nm was measured at 0, 24, 48, and 72 hours using a microplate reader.

### Wound healing experiments

PCa cells were cultured in 6-well plates until confluent. A uniform wound was created by scraping the monolayer with a 20 μL pipette tip, followed by washing with 1× PBS. Wound width was measured at 0 and 24 hours, and the migration rate was calculated as: (Initial wound width - Remaining wound width) / Initial wound width × 100%.

### Statistical analysis

All statistical analyses were conducted using R software (v 4.3.3). Group comparisons for continuous variables were performed with Student's t-test or Wilcoxon rank-sum test, and categorical variables were analyzed using the chi-square test or Fisher's exact test, as appropriate. Correlations between variables were evaluated by Spearman's rank correlation. Survival outcomes were assessed with Kaplan-Meier curves and Cox proportional hazards regression. A two-sided p-value < 0.05 was considered statistically significant.

## Results

### Exploration of TRPM4-related genes and functions in PCa

**Figure 2:**
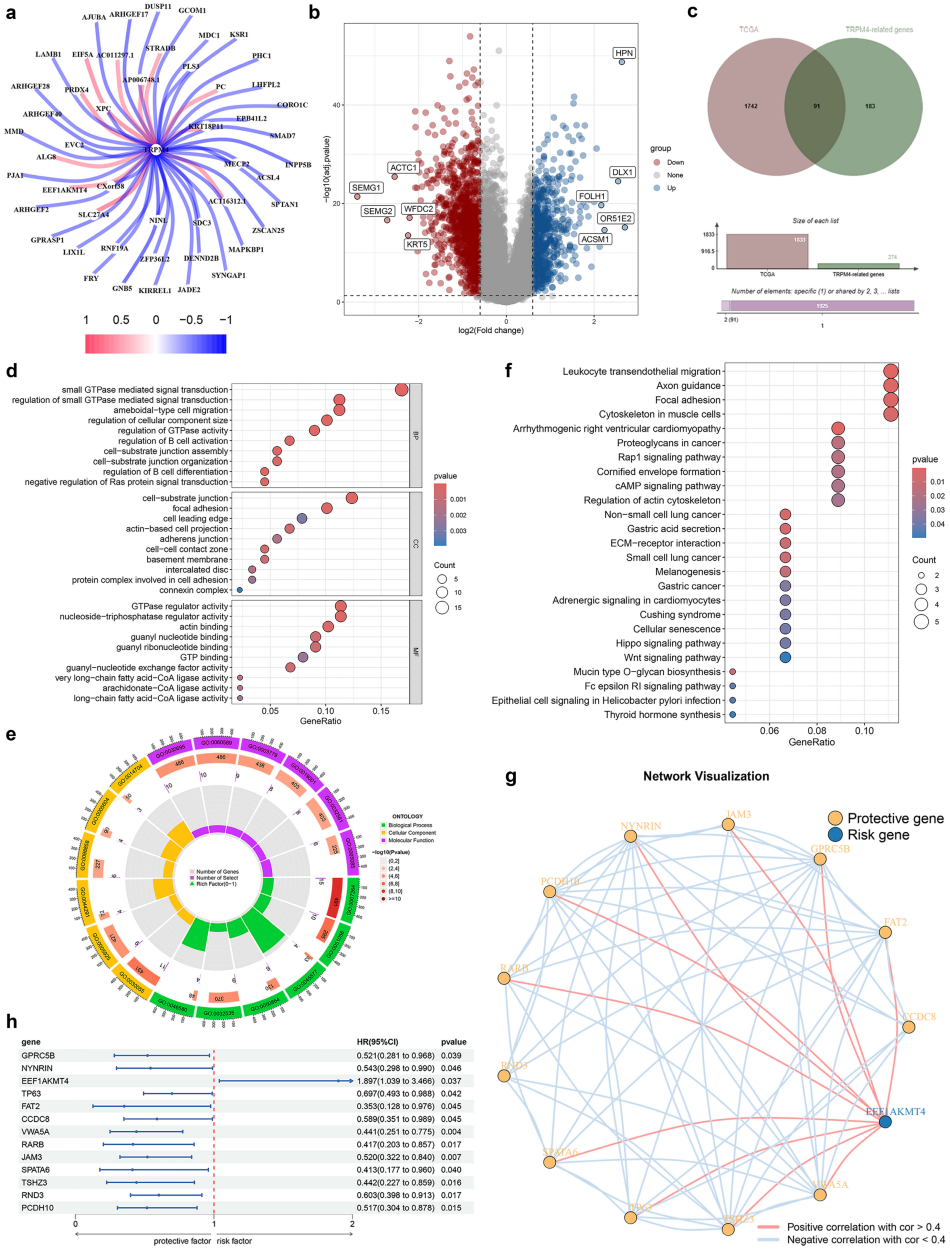
Identification and functional exploration of sodium overload-related genes. (**a**) TRPM4-related gene network diagram. (**b**) volcano plot of PCa vs. normal prostate epithelium. (**c**) Venn diagram. (**d-f**) Functional enrichment of 91 common genes: (**d, e**) GO terms and (**f**) KEGG pathways. (**g**) Network diagram of genes associated with PCa prognosis. (**h**) Prognostic forest plot of disease-free survival (DFS)-associated genes.

Based on our screening criteria, we identified 274 TRPM4-associated genes as potential mediators of TRPM4 (**Fig. 2a**; **Table S2**). GO enrichment analysis indicated their involvement in small GTPase-mediated signal transduction, cell-substrate junction assembly, and GTPase regulator activity (**Fig. S1a, b**). KEGG analysis further linked these genes to the Ras signaling pathway and protein digestion/absorption (**Fig. S1c, d**).

Analysis of TCGA data revealed 1,833 differentially expressed genes in PCa versus normal tissues (**Fig. 2b**). Cross-referencing yielded 91 overlapping genes (**Fig. 2c**), whose GO terms again emphasized small GTPase signaling and cell-matrix adhesion (**Fig. 2d, e**). KEGG pathway analysis highlighted ECM-receptor interaction, Rap1 signaling, cAMP signaling, and the Hippo pathway (**Fig. 2f**). Among these 91 genes, 13 showed association with DFS (**Fig. 2h**), with EEF1AKMT4 representing a risk factor and the remaining 12 genes exhibiting protective effects (**Fig. 2g**).

### Constructing the TRSM through machine learning algorithms

**Figure 3:**
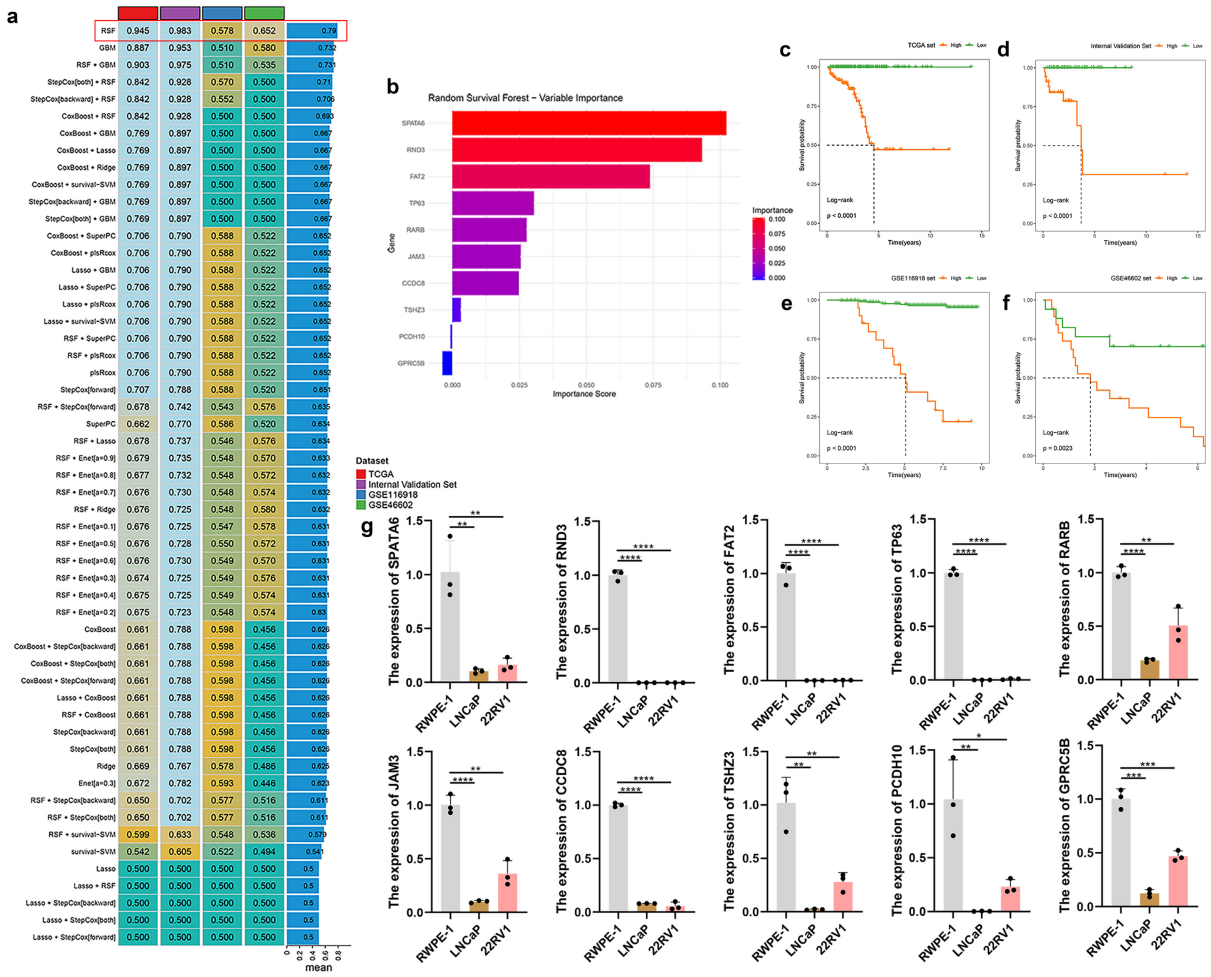
Developing and validating optimal models through machine learning algorithms, and verifying the expression of model genes in normal prostate epithelium cell and various PCa cell lines (LNCap and 22RV1) via RT-PCR. (**a**) A total of 47 predictive models were constructed and further computed in all validation datasets (GSE116918 and GSE46602). (**b**) The importance bar charts for various genes in the random forest model. (**c-f**) Kaplan-Meier curves of disease-free survival (DFS) according to the model scores in (**c**) TCGA training set, (**d**) Internal Validation Set, (**e**) GSE116918 validation set, and (**f**) GSE46602 validation set. (**g**) The expression of 10 modeled genes in two PCa cells (LNCaP and 22RV1) and a normal prostate epithelial cell (RWPE-1).

Multiple machine learning models were trained on the TCGA-PRAD cohort and externally validated using an internal validation set as well as two external validation sets (GSE46602 and GSE116918). Among the models evaluated, the RSF algorithm achieved the highest predictive performance, yielding C-index values of 0.945 in the TCGA training set, 0.983 in the internal validation set, 0.578 in GSE116918, and 0.652 in GSE46602 (**Fig. 3a**). Within the RSF model, SPATA6 was identified as the most influential feature (**Fig. 3b**). Kaplan-Meier analysis demonstrated significantly worse survival among high-risk patients across all three cohorts (TCGA: p < 0.001; internal validation set: p < 0.001; GSE116918: p < 0.001; GSE46602: p = 0.002; **Fig. 3c-f**). qRT-PCR analysis revealed significant downregulation of all ten signature genes—SPATA6, RND3, FAT2, TP63, RARB, JAM3, CCDC8, TSHZ3, PCDH10, and GPRC5B—in LNCaP and 22RV1 PCa cells compared to normal prostate epithelial cells (**Fig. 3g**). Consistently, expression of all ten genes was reduced in TCGA-PRAD tumor samples relative to normal tissues (**Fig. S2a-j**). Receiver operating characteristic (ROC) analysis showed area under the curve (AUC) values exceeding 0.75 for each gene, indicating strong diagnostic potential (**Fig. S2k-t**).

### Clinical characteristics of different TRSM subtypes and construction of nomograms

**Figure 4:**
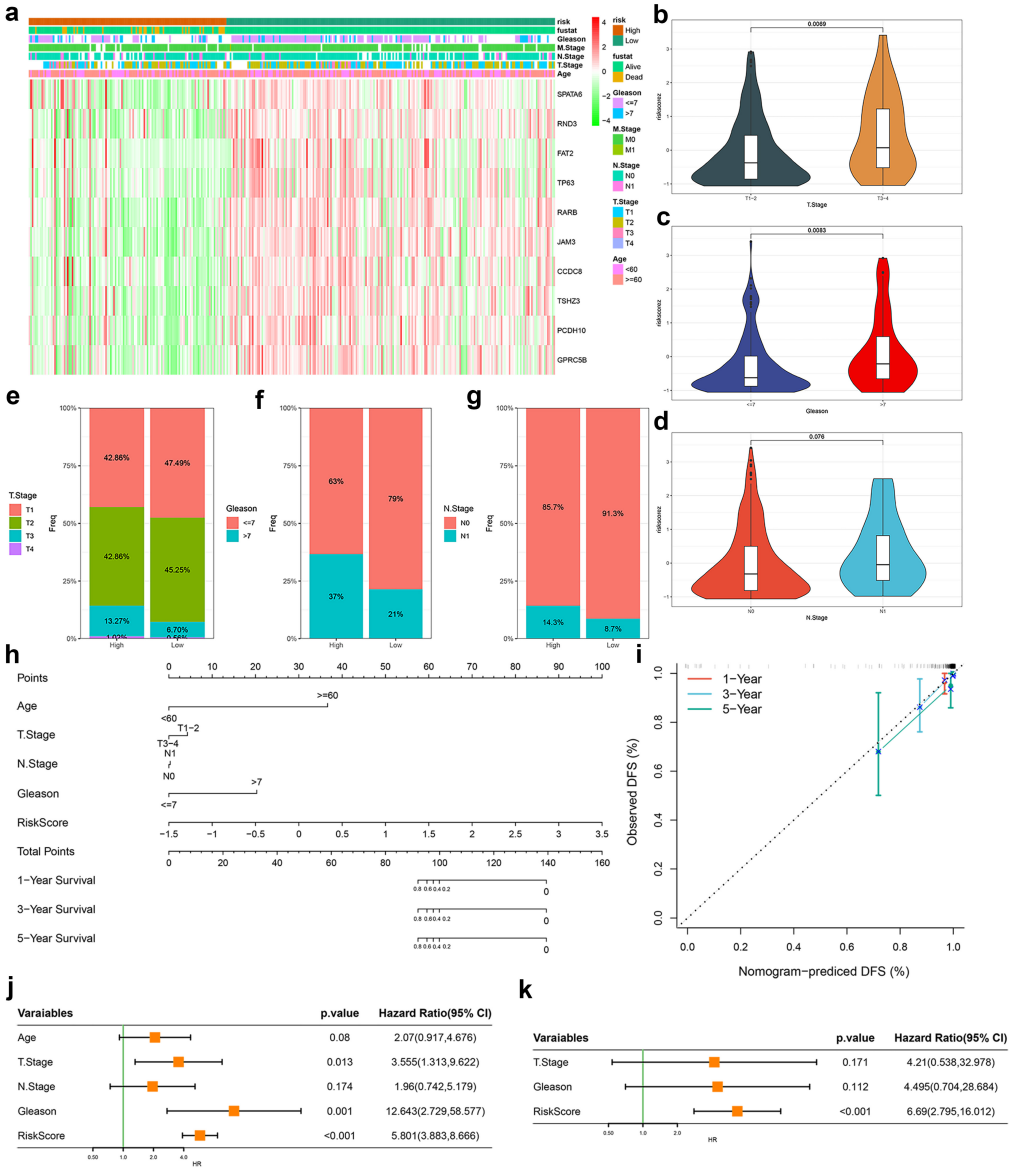
Evaluation of the TRPM4-related signatures model (TRSM). (**a**) Clinical feature and key gene expression distribution by risk score. (**b-d**) Differences in risk scores between patients grouped according to (**b**) T-stage, (**c**) Gleason score, and (**d**) N-stage. (**e-g**) The proportion of (**e**) T stage, (**f**) Gleason score, and (**g**) N-stage in TRSM risk subgroups. (**h**) Nomogram integrating TRSM with clinical parameters. (**i**) Nomogram calibration (1/3/5-year DFS). (**j-k**) Forest plots for (**j**) univariate and (**k**) multivariate Cox regression.

Based on TRSM scores derived from our predictive model, we stratified TCGA-PRAD patients into high- and low-risk groups. A clinical feature heatmap revealed distinct distributions of age, T/N/M stage, and Gleason score between these groups (**Fig. 4a**). Violin plots showed significantly elevated TRSM scores in patients with T3-4 disease compared to T1-2 (p = 0.007; **Fig. 4b**), and in those with Gleason score >7 versus ≤7 (p = 0.009; **Fig. 4c**). Although N1 patients displayed higher median TRSM scores than N0 patients, this trend did not reach statistical significance (p = 0.076; **Fig. 4d**). Stacked bar charts further illustrated the distribution of T stage, Gleason score, and N stage across risk categories (**Fig. 4e-g**). To evaluate clinical applicability, we built a prognostic nomogram integrating TRSM with key clinical variables—age, T stage, N stage, and Gleason score—enabling individualized survival prediction (**Fig. 4h**). Calibration curves demonstrated strong agreement between predicted and observed outcomes across time points (**Fig. 4i**). Both univariate and multivariate Cox regression analyses established TRSM as an independent prognostic factor (HR = 6.690, 95% CI = 2.795-16.012, p < 0.001; **Fig. 4j, k**).

### Mutation characteristics of different TRSM subtypes

**Figure 5:**
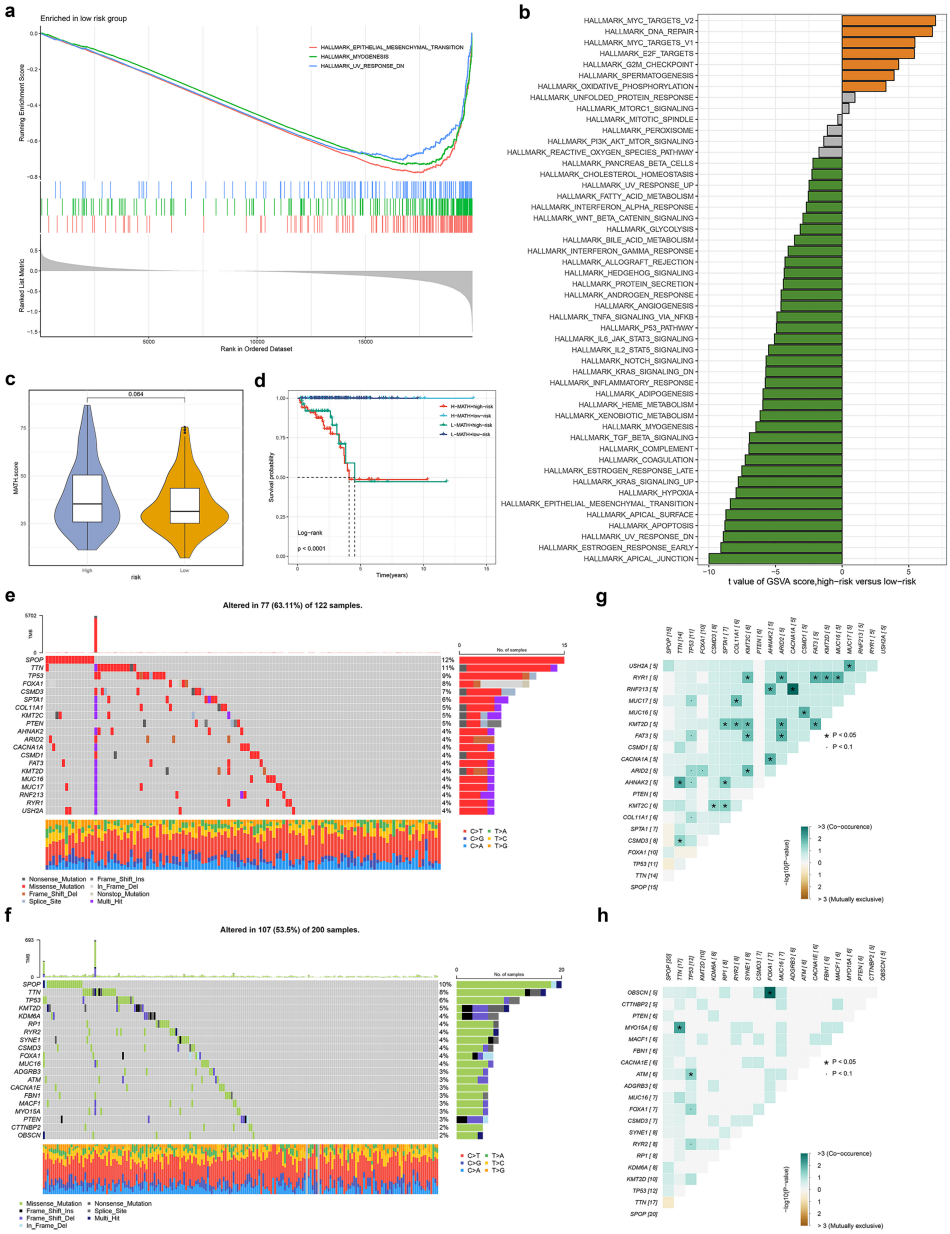
The transcriptome features and Genetic alterations related to TRSM in different risk groups. (**a**) GO terms enriched in low-risk groups analyzed by GSEA. (**b**) Differences in hallmark pathway activities between the high and low-risk groups scored by GSVA. (**c**) The violin plot shows the difference in MATH scores between the high- and low-risk groups. (**d**) Kaplan-Meier curve analysis for RFS of PCa by combining the MATH score and the TRSM risk score. (**e, f**) The waterfall plot of the somatic mutation landscape in (**e**) high- and (**f**) low-risk groups. (**g, h**) Heatmaps showing the association of co-occurrence and exclusive mutation in (**g**) high- and (**h**) low-risk groups.

To investigate potential mechanisms underlying TRSM stratification, we performed GSEA analysis, which revealed significant enrichment of epithelial-mesenchymal transition, myogenesis, and TNFα signaling via NFκB in the low-risk group (**Fig. 5a, S3a**). GSVA further confirmed differential pathway activity between risk subgroups (**Fig. 5b**). We quantified intratumoral heterogeneity using the Mutation Allele Tumor Heterogeneity (MATH) score, observing a trend toward higher heterogeneity in high-risk patients, though this did not reach statistical significance (**Fig. 5c**). Patients classified as both high-risk and high-MATH exhibited significantly worse prognosis (**Fig. 5d**). Mutational landscape analysis revealed distinct profiles between high- and low-risk groups (**Fig. 5e, f**). Examination of co-occurring and mutually exclusive mutations among the top 20 mutated genes showed a greater frequency of co-occurring genetic alterations in the high-risk subgroup (**Fig. 5g, h**).

### Characteristics of immune landscape between different TRSM subtypes

**Figure 6:**
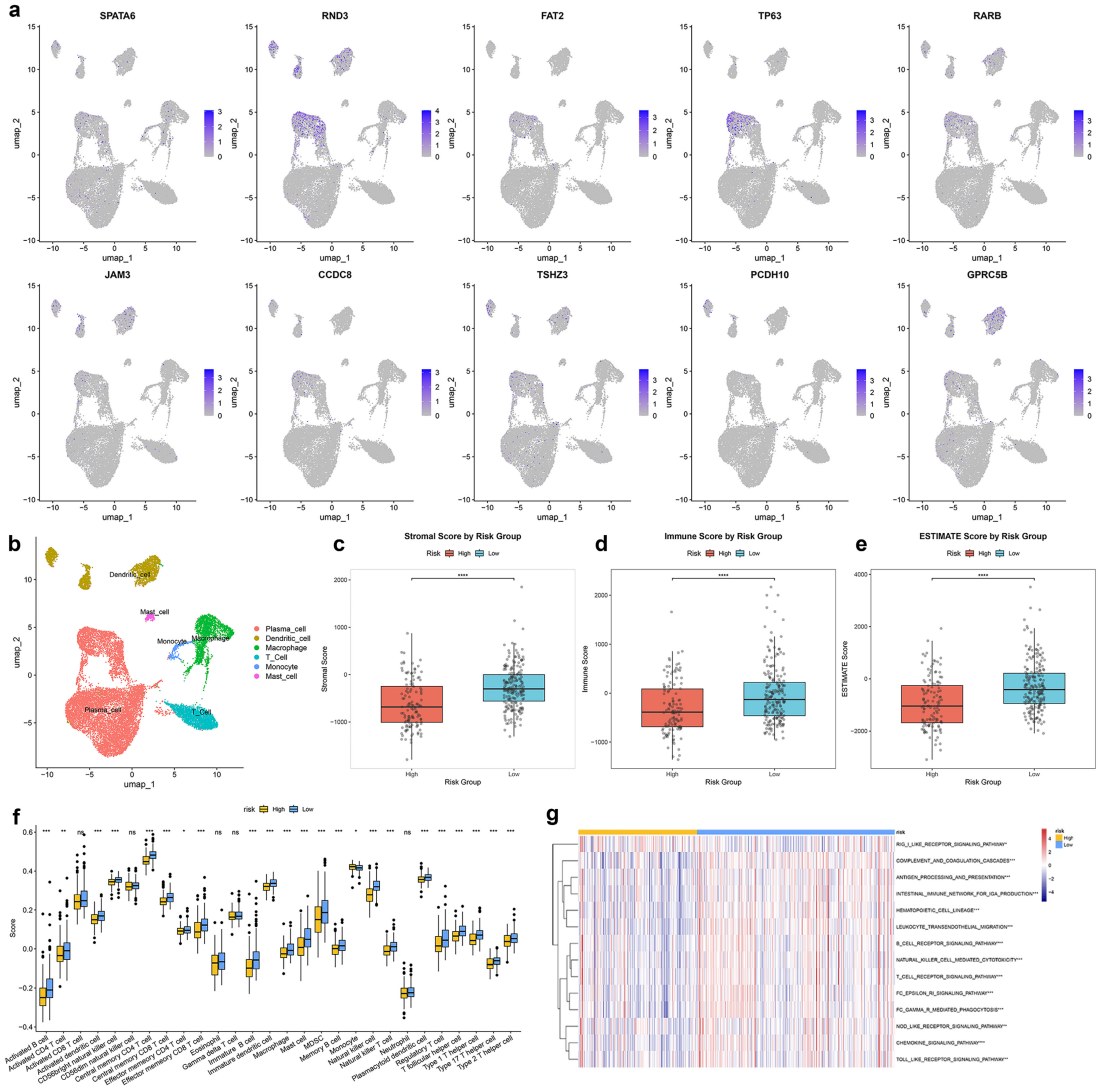
The relationship of TRSM with single-cell characteristics and immune landscape. (**a**) Expression of SPATA6, RND3, FAT2, TP63, RARB, JAM3, CCDC8, TSHZ3, PCDH10, and GPRC5B in various cell types analyzed by single-cell data analysis. (**b**) UMAP visualization of annotated cell types. (**c-e**) Immune microenvironment analysis using the ESTIMATE algorithm based on TRSM risk score stratification: (**c**) Stromal score, (**d**) Immune score, and (**e**) Estimate score. (**f**) Quantification of the abundance of each immune cell type between high- and low- risk groups according to the ssGSEA algorithm. (**g**) Differential immune pathway activity between high- and low- risk groups.

Single-cell transcriptomic analysis identified major cell subpopulations in the prostate tumor microenvironment (**Fig. 6b**), with the ten TRSM signature genes exhibiting distinct cell-type-specific expression patterns (**Fig. 6a**). ESTIMATE analysis indicated that the high-risk group exhibited tumor purity with lower ImmuneScores and ESTIMATE scores (**Fig. 6c-e**). Spearman correlation analysis identified multiple immune cell types significantly associated with TRSM stratification, including reduced infiltration of activated B cells, activated CD4⁺ T cells, and macrophages in high-risk patients (**Fig. 6f**). Further analysis confirmed associations between the ten key genes and levels of immune infiltration (**Fig. S3c**). Immune-related pathway analysis also revealed significant enrichment differences between risk subgroups (**Fig. 6g**). The distribution of tumor-infiltrating immune cells was further characterized using CIBERSORT and ssGSEA, consistently demonstrating distinct immune landscapes between high- and low-risk patients (**Fig. S3b, d**).

### Prediction of immunotherapy response and drug sensitivity

**Figure 7:**
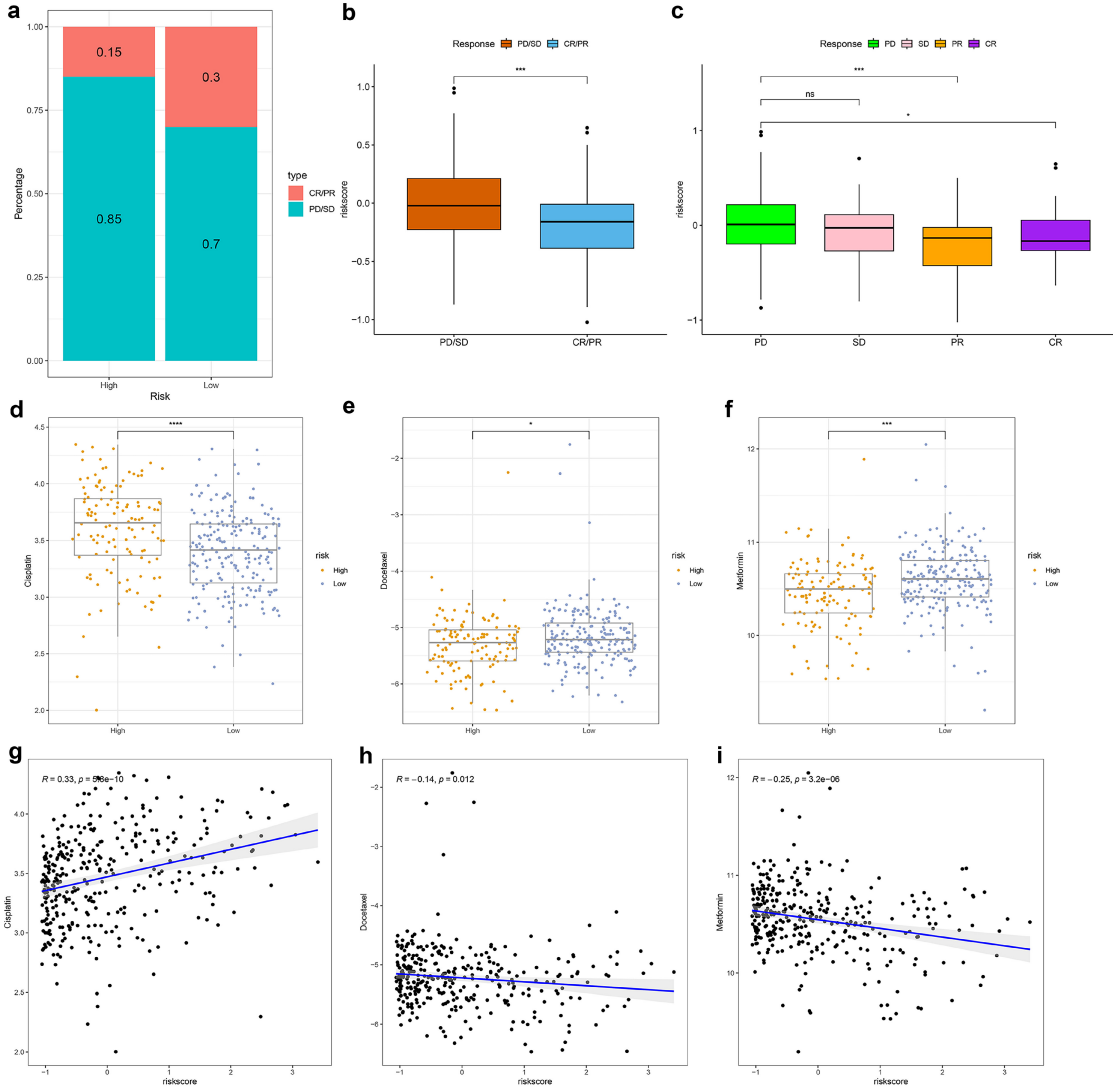
Identification of Immunotherapy sensitivity and the association between the TRSM and drug sensitivity. (**a-b**) IMvigor210 cohort: (**a**) Response rates and (**b**) risk score distribution by treatment outcome. (**c**) Box plot depicting the difference in risk scores between patients with CR, PR, SD, and PD in IMVigor210 cohort. (**d-f**) A comparison of the sensitivity to three drugs, including (**d**) Cisplatin, (**e**) Docetaxel, and (**f**) Metformin in different risk groups. (**g-i**) The relationship between the risk score and the half-maximal inhibitory concentration (IC50) of small molecule drugs, including (**g**) Cisplatin, (**h**) Docetaxel, and (**i**) Metformin.

In the IMvigor210 cohort, high-risk TRSM patients showed a significantly lower proportion of complete or partial responses (CR/PR) compared to the low-risk group (**Fig. 7a**). Consistently, CR/PR patients had significantly lower TRSM risk scores than those with stable or progressive disease (SD/PD) (**Fig. 7b, c**).

Drug sensitivity analysis revealed distinct response patterns between risk subgroups: high-risk patients displayed increased resistance to cisplatin but greater sensitivity to docetaxel and metformin (**Fig. 7d-f**). Correlation analysis further supported these findings, showing a positive association between TRSM risk score and cisplatin IC₅₀ (R = 0.33, p < 0.001), and negative correlations with docetaxel (R = -0.14, p = 0.012) and metformin (R = -0.25, p < 0.001) IC₅₀ values (**Fig. 7g-i**).

### The effect of SPATA6 on PCa cells *in vitro*

**Figure 8:**
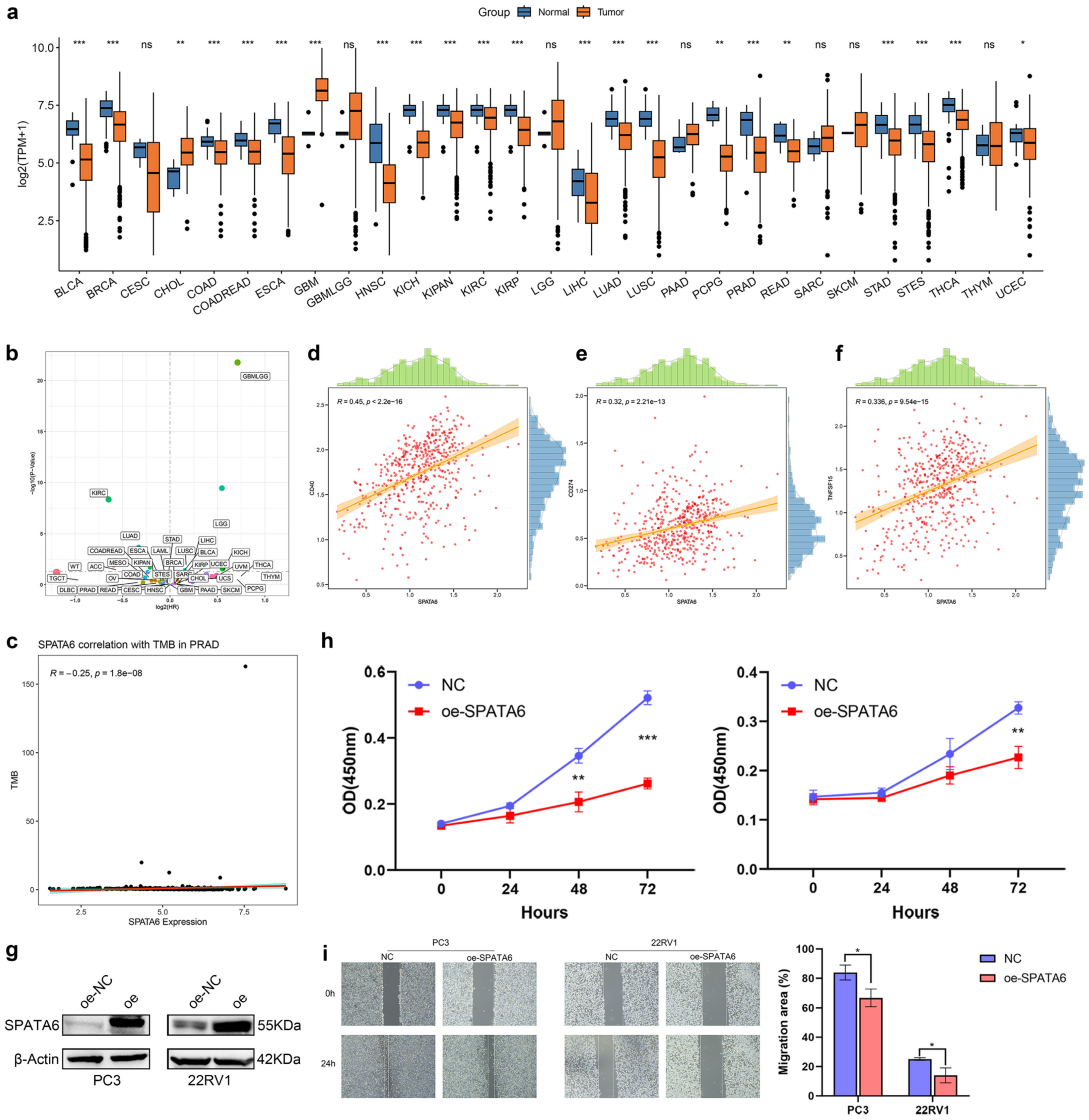
Pan-cancer analysis and functional validation of SPATA6. (**a**) Boxplot of SPATA6 expression across 29 cancer types in the TCGA database. (**b**) Scatter plot of SPATA6 and pan-cancer prognosis (Disease-free survival). (**c**) Scatter plot showing the correlation between SPATA6 and the PCa tumor microenvironment. (**d-f**) Scatter Plot of SPATA6 Correlation with Three Immune Checkpoints: (**d**) CD40, (**e**) CD274, and (**f**) TNFSF15. (**g**) Expression level of SPATA6 was significantly up-regulated in oe-SPATA6 cells. (**h**) CCK-8 analysis of the impact of SPATA6 overexpression on PCa cell growth. (**i**) Wound healing assay of the impact of SPATA6 overexpression on PCa migration.

Pan-cancer analysis revealed consistent downregulation of SPATA6 in three major urologic malignancies: bladder cancer, kidney cancer, and PCa (**Fig. 8a**). Analysis of PFS across cancer types indicated tumor-specific associations between SPATA6 expression and clinical outcomes (**Fig. 8b**). In PCa, SPATA6 levels showed a significant negative correlation with tumor microenvironment scores (**Fig. 8c**). Further immune profiling revealed positive correlations between SPATA6 expression and immune checkpoint molecules CD40, CD274 (PD-L1), and TNFSF15 (**Fig. 8d-f**). Functional studies demonstrated that SPATA6 overexpression in PC3 and 22RV1 PCa cells significantly suppressed both proliferative capacity and cell migration (**Fig. 8g-i**).

## Discussion

Based on TRPM4-associated molecular features, we developed a computational framework that integrated multiple machine learning algorithms and ultimately selected the RSF method to establish a novel prognostic signature. This model effectively stratified patients into distinct risk categories, demonstrated robust predictive performance for survival outcomes across multiple cohorts, and was further validated for its clinical utility. Furthermore, TRSM risk stratification revealed significant differences in immunotherapy response and sensitivity to conventional chemotherapeutic agents. Our study provides association and bioinformatics evidence suggesting a potential link between the TRSM genome, sodium imbalance, and TRPM4-related signaling, but does not provide direct mechanistic proof. In summary, the TRSM model may offer a reasonable framework for personalized risk assessment and treatment selection in PCa.

Functional enrichment analysis of the 91 overlapping genes revealed a strong association with energy metabolism pathways. Notably, among small GTPases, the Ras and Rho families emerged as particularly relevant. Ras, a well-characterized oncogene, drives sustained proliferation, survival, and metabolic reprogramming in PCa through frequent mutational activation 22. Importantly, Ras pathway hyperactivation is more commonly observed in advanced CRPC 23, 24. While one study has suggested possible crosstalk between TRPM4 and Ras 25, whether this interaction operates in PCa or relates to sodium overload remains an open question. Dysregulation of the Hippo signaling pathway also contributes to tumorigenesis. Prior work indicates that TRPM4-mediated calcium signaling can activate downstream kinases such as Rho-associated coiled-coil-containing protein kinase (ROCK) and protein kinase C (PKC) 10, 26, which in turn phosphorylate and modulate Hippo pathway components. However, existing studies have centered almost exclusively on calcium—not sodium—as the ionic mediator. Whether sodium influx through TRPM4 influences the Hippo pathway remains unexplored. The link between TRPM4 and cAMP signaling is both direct and mechanistically well-defined, primarily involving the cAMP sensor EPAC, which stimulates TRPM4 channel activity and promotes burst firing behavior 10, 27. Nevertheless, the functional relevance of this axis in PCa, and its potential connection to sodium overload, has yet to be established. Emerging evidence indicates that the TGF-β/SMAD pathway and EMT are key regulators in prostate pathophysiology 28, 29. Given their potential crosstalk with sodium death-related signaling, exploring these connections may deepen our understanding of the molecular architecture underlying PCa.

The TRSM, constructed via the RSF algorithm, incorporates ten key genes: SPATA6, RND3, FAT2, TP63, RARB, JAM3, CCDC8, TSHZ3, PCDH10, and GPRC5B. Notably, the C-index for GSE116918 was modest at 0.578. It is worth noting that patients in this dataset had undergone prior radiotherapy and androgen deprivation therapy, which may introduce bias and thus affect model accuracy. While SPATA6 is recognized for its role in spermatogenesis 30, its function in cancer remains poorly defined. Our pan-cancer analysis revealed consistent downregulation of SPATA6 across multiple urologic malignancies, suggesting potential tumor-suppressive activity. Functional studies in PCa cells confirmed that SPATA6 overexpression suppresses proliferation, supporting this hypothesis. Notably, SPATA6 has been reported to interact with the androgen receptor (AR) 31, prompting further interest in its potential hormone-related mechanisms in PCa. RND3, a GTP-binding protein lacking GTPase activity, was identified as an NF-κB2/p52 target in PCa, though its mechanistic contributions remain unclear 32. TP63, also downregulated in PCa as validated in our data, participates in transcriptional networks involving S100A14 and the lncRNA CTBP1-AS, and modulates enhancer methylation and genomic instability via the TP63-TRIM29 axis 33-35.

RARB expression is reduced in PCa, with studies consistently linking RARB promoter hypermethylation to aggressive clinicopathological features and poor prognosis 36-40. We can understand DNA methylation as a “switch” for genes: adding a methyl group to the promoter region of a gene leads to its silencing. This epigenetic silencing is facilitated through collaboration with EZH2 during tumor progression 41. JAM3 has also emerged from methylation profiling as a gene of interest in the context of PTEN loss 42, 43. Although TSHZ2 acts as a tumor suppressor in PCa, it appears not to be regulated by methylation in this malignancy 44. In contrast, PCDH10 methylation is strongly associated with elevated PSA, advanced stage, high Gleason score, lymph node metastasis, and biochemical recurrence 45, 46. A notable pattern emerging from these genes is the importance of epigenetic regulation, particularly DNA methylation. Although there is currently no direct evidence indicating a correlation between sodium overload and the dynamics of methylation, literature suggest that sodium pressure and inflammatory/oxidative stress pathways can all affect epigenetic regulation 47. Based on this indirect evidence, we cannot help but speculate that sodium overload may cause changes in epigenetic balance through inflammatory or oxidative stress-related mechanisms. However, given the lack of direct experimental verification in current studies, this mechanism assumption remains speculative and requires further research. Future studies are warranted to directly investigate whether TRPM4-mediated sodium overload influences the DNA methylation status of specific target genes—particularly those implicated in inflammatory or oxidative stress responses—thereby establishing a causal link between ionic perturbation and epigenetic remodeling.

Stratification of PCa patients by TRSM score revealed that high-risk patients display a broadly immunosuppressed tumor microenvironment. Specifically, we observed reduced infiltration of both cytotoxic CD4⁺ T cells—which can eliminate tumor cells via perforin-granzyme and FAS/FASL pathways 48, 49—and regulatory T cells (Tregs), which are critical for maintaining immune tolerance 50. Consistent with these findings, the high-risk group showed poorer predicted responses to immunotherapy, indicating that TRSM reflects immunologically cold tumors and may help identify patients less likely to benefit from immune checkpoint inhibition. While sodium overload has been linked to immune modulation, its role in PCa remains unclear. High salt conditions are known to promote pro-inflammatory Th17 differentiation, yet may also disrupt overall immune homeostasis 51. In cancer, such disruption could impair antitumor immunity. Sodium ions may further amplify inflammatory signaling via Na⁺/K⁺-ATPase-mediated pathways, potentially contributing to T cell exhaustion under chronic stimulation 52. We thus propose that sodium overload in PCa may alter the balance of CD4⁺ T cell subsets, favoring an immunosuppressive state. Future studies should delineate how sodium signaling specifically influences distinct T cell populations in the tumor microenvironment. It is worth noting that the IMvigor210 cohort consists of patients with urothelial carcinoma, not PCa. Although this cohort has been widely used as a benchmark for predicting immunotherapy responses beyond bladder cancer, the tumor immune microenvironment in PCa differs from that of urothelial carcinoma in several key respects. Accordingly, future validation in a dedicated PCa immunotherapy cohort is warranted.

Docetaxel serves as a first-line chemotherapy for CRPC, exerting antitumor effects through microtubule stabilization, suppression of AR nuclear translocation, and downregulation of AR and its splice variants 53-56. Intriguingly, TRSM-classified high-risk patients showed heightened sensitivity to docetaxel, indicating its potential therapeutic relevance for this subgroup. TRPM4 is a non-selective cation channel that requires considerable ATP consumption for its activity, with ion homeostasis being maintained via the Na⁺/K⁺-ATPase pump 13. This increased metabolic demand may render high-risk tumor cells particularly susceptible to agents that disrupt metabolic pathways or cytoskeletal integrity. Docetaxel, for example, acts not only by preventing mitosis but also by interfering with intracellular transport and signal transduction 57. Consequently, in cells characterized by high ion flux, the already stressed cytoskeleton may be predisposed to catastrophic damage following docetaxel exposure. In contrast, cisplatin is seldom used in PCa outside the context of treatment-emergent neuroendocrine histology 58. Although a PD-1 blockade plus platinum-based regimen achieved a 43% response rate in neuroendocrine PCa 59, our data suggest that high-risk patients may exhibit cisplatin resistance. Metformin has been linked to reduced PCa risk, potentially through ameliorating hyperinsulinemia-driven tumorigenesis or via direct cellular effects 60. Its activity in PCa—especially among patients with diabetes or metabolic syndrome—is supported by several studies 61, 62. Furthermore, the features related to TRPM4 are relatively abundant in pathways such as cAMP signal transduction and small GTPase activity, both of which have been proven to interact with AMPK signal transduction. Therefore, we speculate that metformin, as an AMPK activator 63, may produce different effects depending on our risk stratification. It is worth noting that these predictions were generated using the "pRRophetic" software package, which is a computational method for inferring drug responses based on transcriptome data 64. Although this approach provides valuable insights into potential drug efficacy and is helpful for high-throughput screening, it cannot replace direct *in vitro* or *in vivo* experimental validation. The results of this computational analysis should be regarded as the basis for proposing hypotheses, and further experiments and clinical validation are required to confirm them.

Despite the promising findings of this study, several limitations should be acknowledged. Firstly, although our analysis was initiated from TRPM4, the number of well-characterized sodium overload-associated genes remains limited. Further mechanistic studies are needed to experimentally validate the relationship between TRSM and sodium overload-induced cell death. Secondly, although TRSM has undergone rigorous evaluation using TCGA data, the internal validation set, and external independent validation cohorts, its clinical utility still needs to be further confirmed in large-scale, prospective, multicenter studies. Thirdly, it should be noted that the single-cell data was used solely to assess the expression of core genes in the TRSM model and did not contribute to the construction of our model. In the future, we will need to further utilize and expand upon the single-cell data to conduct more in-depth research. Finally, although we established a functional role for SPATA6 in PCa, the biological contributions of other signature genes in the model await experimental clarification.

## Conclusion

In this study, we constructed a PCa diagnostic model—TRSM—by targeting TRPM4, a central mediator of sodium overload. Validation across multiple cohorts demonstrated that TRSM robustly predicts patient prognosis and informs personalized therapeutic selection, supporting its potential clinical utility. Moreover, our integrated genomic and single-cell transcriptomic analyses provide new mechanistic insights into PCa development and progression. Future studies should further elucidate the functional relationship between TRPM4 and sodium overload within the prostate tumor microenvironment.

## Supplementary Material

Supplementary figures and tables.
