# Supplementary material for: Integrative Machine Learning Framework Revealing TRPM4-Associated Signatures and Identifying SPATA6 as a Potential Biomarker in Prostate Cancer
Source: J Cancer. 2026 May 11;17(5):1002–17. doi: 10.7150/jca.129356 (PMC13189831; doi:10.7150/jca.129356)
Supplement: Supplementary file 1 — Supplementary figures and tables. [file jcav17p1002s1.pdf]

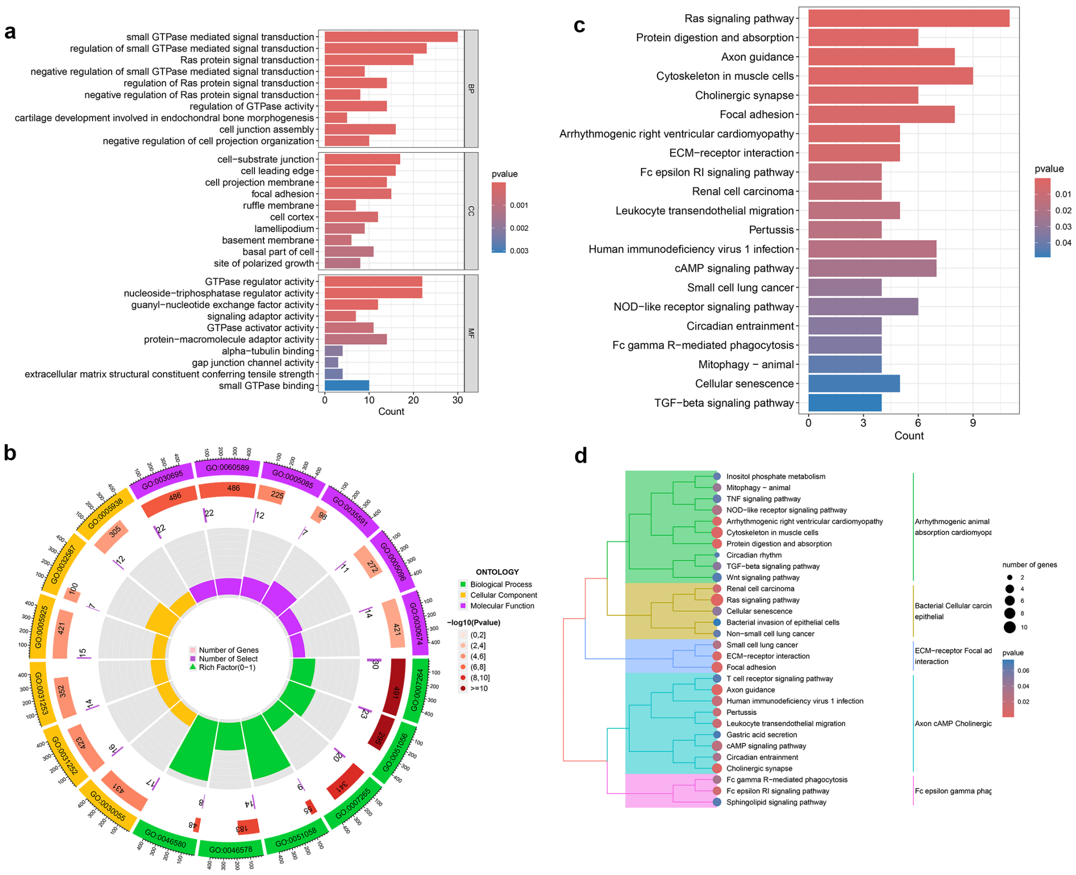

Fig. S1 Functional enrichment analysis of 274 genes associated with TRPM4. (**a-b**) Bar chart and circle graph in GO Analysis. (**d-e**) Bar chart and tree plot in KEGG pathways.

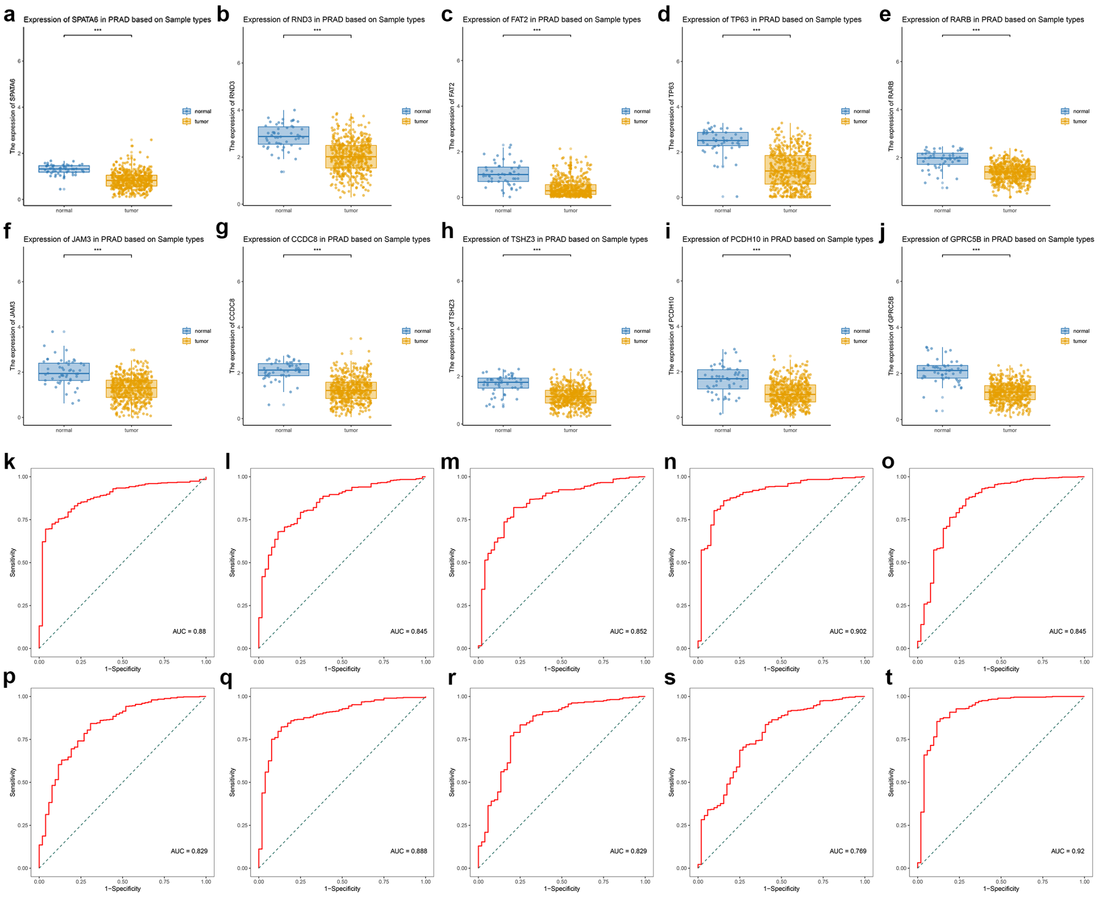

Fig. S2 Expression and diagnostic characteristics of 10 key genes in the TRSM. (**a-j**) Boxplot of expression levels for 10 key genes: (**a**) SPATA6, (**b**) RND3, (**c**) FAT2, (**d**) TP63, (**e**) RARB, (**f**) JAM3, (**g**) CCDC8, (**h**) TSHZ3, (**i**) PCDH10, and (**j**) GPRC5B. (**k-t**) ROC curve of 10 key genes: (**k**) SPATA6, (**l**) RND3, (**m**) FAT2, (**n**) TP63, (**o**) RARB, (**p**) JAM3, (**q**) CCDC8, (**r**) TSHZ3, (**s**) PCDH10, and (**t**) GPRC5B.

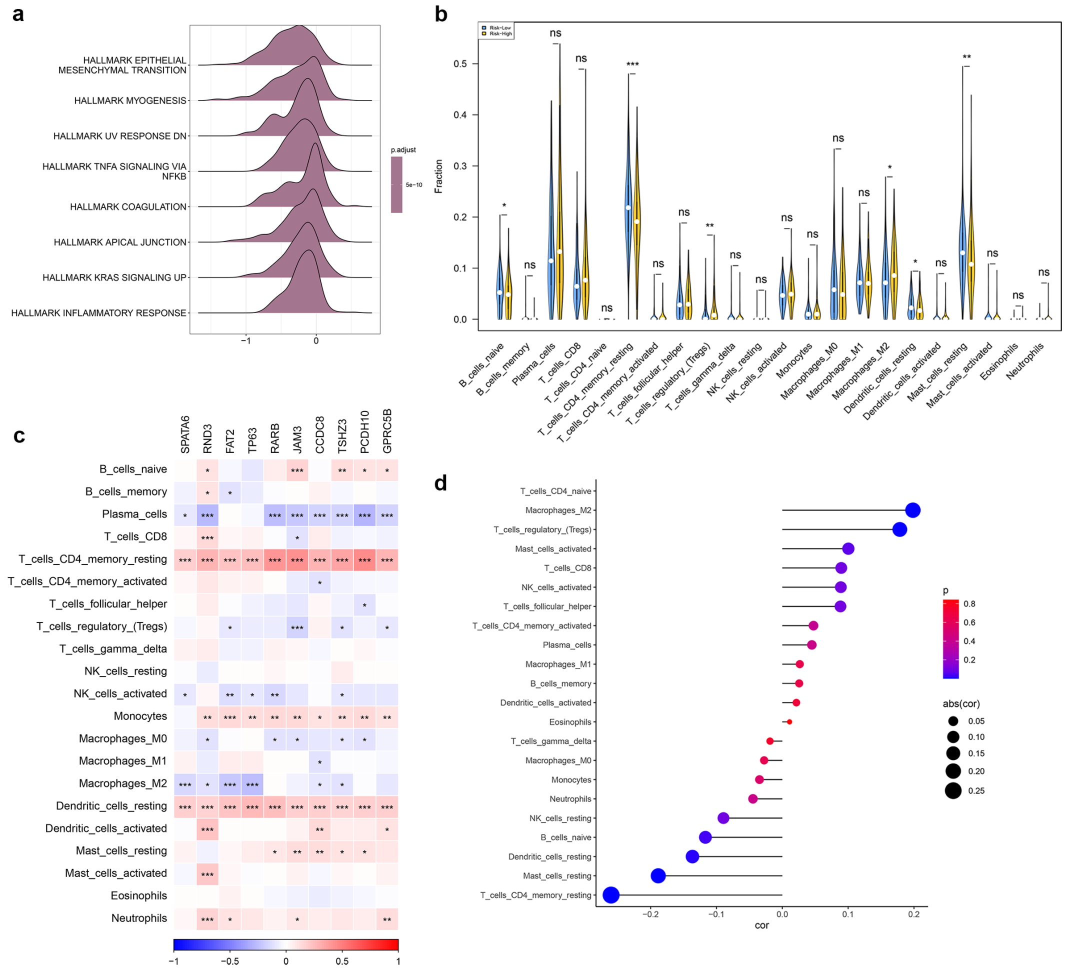

Fig. S3 Supplementary analysis of the relationship between TRSM and immune cell infiltration. (**a**) Ridge plot showing GO terms enriched in low-risk groups. (**b**) Quantification of the abundance of each immune cell type between high- and low- risk groups according to the CIBERSORT algorithm. (**c**) Association between TME-infiltrated cells and key genes of TRSM. (**d**) The relationship between TME-infiltrated cells and TRSM.

Table S1. Primer sequences of the qRT-PCR.

Genes	Primer sequences
SPATA6-F	GACTCTGCCTATGACAGTGACC
SPATA6-R	GCTGTTCTCAAAGATGGGTCGG
RND3-F	AATCACAGGCAGACGCCAGTGT
RND3-R	CCGACTGTAAAGCTGAGCATTCG
FAT2-F	CACACTGACAGTCATGGTCCGA
FAT2-R	TGGTGTCAGGAACACTTGCCTC
TP63-F	CAGGAAGACAGAGTGTGCTGGT
TP63-R	AATTGGACGGCGGTTCATCCCT
RARB-F	GGTTTCACTGGCTTGACCATCG
RARB-R	CCGTCTGAGAAAGTCATGGTGTC
JAM3-F	GCTGTGCATACAGACGTGGCTA
JAM3-R	TCCTCGTCAGTGCGGATGTAGT
CCDC8-F	GAGTTCAGCGACTTCGAGACCT
CCDC8-R	ATCATCCTCACTCTCCAGGTCG
TSHZ3-F	CACCTACCATCACAACCCTGCT
TSHZ3-R	CGACTTCCTTCTTGACCTCCAC
PCDH10-F	TCTCCAACGGAAGCATTTTGTCC
PCDH10-R	CTATGTCGGCTTCCTGGAATGC
GPRC5B-F	CAGGAGAACACGCCCAACTACT
GPRC5B-R	GGAAATCCTGCTGTTCGGAGAG

Table S2. 274 genes associated with TRPM4 in PCa

Sodium-related	Gene	Cor	pvalue
TRPM4	SLC27A4	0.567	4.80259479725518e-44
TRPM4	GNB5	-0.528	1.90255025988146e-37
TRPM4	EIF5A	0.510	1.25791132545741e-34
TRPM4	INPP5B	-0.492	6.41638262827615e-32
TRPM4	ARHGEF17	-0.481	2.06613762878772e-30
TRPM4	PRDX4	0.476	9.38025279315996e-30
TRPM4	MECP2	-0.475	1.40067102029738e-29
TRPM4	ACSL4	-0.471	4.85885979396078e-29
TRPM4	AC116312.1	0.469	7.70581563371481e-29
TRPM4	ZSCAN25	-0.461	8.46915622789717e-28
TRPM4	DENND2B	-0.449	2.62565105211558e-26
TRPM4	ARHGEF40	-0.446	5.87321557339713e-26
TRPM4	ZFP36L2	-0.443	1.5698712978349e-25
TRPM4	AJUBA	-0.442	1.81704126549032e-25
TRPM4	MAPKBP1	-0.442	2.22842610946571e-25
TRPM4	XPC	-0.441	2.61191020834453e-25
TRPM4	SMAD7	-0.441	2.63015608648052e-25
TRPM4	PHC1	-0.439	4.13119778820147e-25
TRPM4	GPRASP1	-0.437	8.05561820536755e-25
TRPM4	KRT18P11	0.436	9.48951023464386e-25
TRPM4	KIRREL1	-0.433	2.20641112122622e-24
TRPM4	DUSP11	-0.432	2.79075694196167e-24
TRPM4	EPB41L2	-0.432	3.11103425698606e-24
TRPM4	CXorf38	-0.429	6.61178121286852e-24
TRPM4	AP006748.1	0.427	1.11991255139915e-23
TRPM4	RNF19A	-0.426	1.35932235885636e-23
TRPM4	PC	0.426	1.56427897498323e-23
TRPM4	EVC2	-0.426	1.63306215301507e-23
TRPM4	SPTAN1	-0.425	2.00262502645473e-23
TRPM4	EEF1AKMT4	0.424	2.50261193180171e-23
TRPM4	ALG8	0.424	2.64925353973793e-23
TRPM4	AC011297.1	0.423	2.96620687606405e-23
TRPM4	JADE2	-0.423	3.13622779460307e-23
TRPM4	KSR1	-0.422	4.20787818555468e-23
TRPM4	NINL	-0.421	5.31253593750848e-23
TRPM4	ARHGEF2	-0.419	1.01912946100953e-22
TRPM4	GCOM1	-0.418	1.09528851965453e-22
TRPM4	PJA1	-0.416	2.00905544598306e-22
TRPM4	SYNGAP1	-0.415	2.33996122772639e-22
TRPM4	MDC1	-0.415	2.68552583565681e-22
TRPM4	STRADB	0.415	2.89736029797635e-22
TRPM4	FRY	-0.414	3.29747891132629e-22

TRPM4	LIX1L	-0.414	3.35445707587304e-22
TRPM4	LHFPL2	-0.413	3.99627687923218e-22
TRPM4	LAMB1	-0.413	4.33383608332565e-22
TRPM4	ARHGEF28	-0.413	4.57546761277926e-22
TRPM4	PLS3	-0.412	4.88878835983691e-22
TRPM4	MMD	-0.410	9.24194245248216e-22
TRPM4	SDC3	-0.408	1.36209805243546e-21
TRPM4	CORO1C	-0.408	1.4307698951158e-21
TRPM4	NOL4L	-0.407	1.80812779786987e-21
TRPM4	BTN2A1	-0.406	2.17757347431175e-21
TRPM4	ZFP92	-0.406	2.31414665727655e-21
TRPM4	RNF25	0.406	2.46154447592797e-21
TRPM4	C11orf95	-0.405	2.80521073666132e-21
TRPM4	SEMA5A	-0.403	4.51796216742725e-21
TRPM4	NUAK1	-0.403	4.81456396549389e-21
TRPM4	RGL1	-0.402	6.39151427878867e-21
TRPM4	PLCE1	-0.400	9.78120492290413e-21
TRPM4	GNAI2	-0.399	1.18431657364986e-20
TRPM4	GPRC5B	-0.399	1.2124692281218e-20
TRPM4	RABGEF1	0.399	1.33671875653302e-20
TRPM4	TFE3	-0.399	1.35889664308313e-20
TRPM4	IQCA1	-0.399	1.40521090190751e-20
TRPM4	MVB12B	-0.398	1.51866194885357e-20
TRPM4	MSN	-0.397	1.92406987819264e-20
TRPM4	NCKAP5L	-0.396	2.69363699242569e-20
TRPM4	NYNRIN	-0.395	3.04522940940204e-20
TRPM4	CUL1	-0.395	3.29131795783185e-20
TRPM4	PHLDB1	-0.394	4.07327859085311e-20
TRPM4	MFN2	-0.394	4.17586791229855e-20
TRPM4	TMEM126A	0.394	4.23105364892678e-20
TRPM4	ZNF665	-0.393	5.27917023194318e-20
TRPM4	AL354872.1	0.393	5.6646690342674e-20
TRPM4	FUCA2	0.392	6.20353179357004e-20
TRPM4	AHDC1	-0.391	8.98946101129923e-20
TRPM4	ATP1B1	-0.391	9.11076399362634e-20
TRPM4	RNF165	-0.391	9.19712825397802e-20
TRPM4	MECOM	-0.389	1.39949137891954e-19
TRPM4	GJA1	-0.388	1.67931626867217e-19
TRPM4	NME1	0.388	1.72930195174152e-19
TRPM4	PARP3	-0.388	1.84181715165027e-19
TRPM4	EFNB3	-0.386	2.95081480731921e-19
TRPM4	TBC1D1	-0.385	3.45740613481063e-19
TRPM4	HSD3B7	0.384	4.1379330135121e-19
TRPM4	SGSM1	-0.383	5.0669962394844e-19

TRPM4	ITGA3	-0.383	5.10849886021236e-19
TRPM4	DAAM2	-0.381	8.60443828703166e-19
TRPM4	RAB38	-0.381	8.96599992377077e-19
TRPM4	ZNF660	-0.381	9.13926435461231e-19
TRPM4	TP63	-0.381	9.238544761248e-19
TRPM4	PCDH10	-0.379	1.49324126570204e-18
TRPM4	MAP3K12	-0.377	2.15104928433852e-18
TRPM4	ARHGAP23	-0.377	2.16358180103072e-18
TRPM4	ETNK2	-0.377	2.16741555409281e-18
TRPM4	TCF7L1	-0.376	2.44438090732065e-18
TRPM4	ZNF804A	-0.376	2.6376771142634e-18
TRPM4	SYT11	-0.376	2.83273796359869e-18
TRPM4	ID2-AS1	-0.376	2.84604271365808e-18
TRPM4	NRF1	-0.375	2.99049378521226e-18
TRPM4	CELF1	-0.375	3.32546248482617e-18
TRPM4	APOBEC3G	-0.375	3.4706498302239e-18
TRPM4	MET	-0.374	3.86896149160853e-18
TRPM4	PTPRE	-0.373	4.83053003390199e-18
TRPM4	AFAP1L2	-0.373	5.07674052233397e-18
TRPM4	HLA-F-AS1	-0.373	5.13067985865057e-18
TRPM4	SH2B3	-0.372	6.67864257604206e-18
TRPM4	GGPS1	0.372	7.08667859407748e-18
TRPM4	DNAJB2	0.370	9.80341554495117e-18
TRPM4	PLEKHG5	-0.367	1.80378649542129e-17
TRPM4	RASSF5	-0.367	2.07852200376263e-17
TRPM4	DHX35	-0.366	2.10850185013385e-17
TRPM4	CLIP2	-0.366	2.39994453547628e-17
TRPM4	PRPF40B	-0.365	2.79186478609846e-17
TRPM4	ELF4	-0.365	2.8821504528413e-17
TRPM4	OAT	-0.364	3.37659014522518e-17
TRPM4	RND3	-0.364	3.39093242847408e-17
TRPM4	NTN1	-0.364	3.52643609001255e-17
TRPM4	ZBTB46	-0.361	7.16773755189869e-17
TRPM4	INPP5D	-0.359	1.05903059612164e-16
TRPM4	EXT1	-0.359	1.08194040294516e-16
TRPM4	ZFP36L1	-0.359	1.12800362739766e-16
TRPM4	GAB2	-0.357	1.65498391627746e-16
TRPM4	FHOD3	-0.356	2.05546826834885e-16
TRPM4	AOPEP	-0.355	2.3643864380258e-16
TRPM4	RIMKLB	-0.355	2.59122276147453e-16
TRPM4	ARHGAP24	-0.354	2.65210310519252e-16
TRPM4	VWA5A	-0.353	3.2066865693486e-16
TRPM4	ID4	-0.353	3.47467601936247e-16
TRPM4	ENO2	-0.353	3.49481168470192e-16

TRPM4	WDR91	-0.353	3.65386162632946e-16
TRPM4	ADAMTSL3	-0.352	4.10893089818534e-16
TRPM4	AC018521.6	-0.352	4.37040788008688e-16
TRPM4	ITPRIP	-0.351	5.10225642068515e-16
TRPM4	ETS1	-0.351	5.59129548163006e-16
TRPM4	MB21D2	-0.350	6.12978079947696e-16
TRPM4	VASH1	-0.350	6.41091436017294e-16
TRPM4	ARL4C	-0.349	7.829299737646e-16
TRPM4	SLC35B1	0.349	8.57344267523272e-16
TRPM4	TRAF3IP1	-0.348	1.00203578202213e-15
TRPM4	FAT2	-0.348	1.0719720949734e-15
TRPM4	DNM3	-0.347	1.1774607112324e-15
TRPM4	BEST1	-0.347	1.19494589598991e-15
TRPM4	EPS8	-0.347	1.26538843114915e-15
TRPM4	SLC6A6	-0.346	1.34205589231201e-15
TRPM4	COL17A1	-0.346	1.45295016537463e-15
TRPM4	AP1M2	0.346	1.50977574724545e-15
TRPM4	RARB	-0.345	1.61867673984105e-15
TRPM4	WWC2-AS2	-0.345	1.63025195812455e-15
TRPM4	LTBP3	-0.345	1.90997736852334e-15
TRPM4	ARAP1	-0.344	2.25911717860451e-15
TRPM4	VAV3	-0.343	2.39314577734096e-15
TRPM4	ITPRIPL1	-0.343	2.43359361865212e-15
TRPM4	DBNDD1	0.342	2.93932954825978e-15
TRPM4	ID2	-0.342	3.439568605187e-15
TRPM4	INTS6L	-0.341	3.58834426589871e-15
TRPM4	FLJ20021	0.341	3.59433283909746e-15
TRPM4	RAB7B	-0.341	3.989827772535e-15
TRPM4	APBB1	-0.340	5.22010998839035e-15
TRPM4	DAB2IP	-0.339	6.19882922846045e-15
TRPM4	RUSC2	-0.338	6.52629767311523e-15
TRPM4	SPRY1	-0.338	6.90791120232301e-15
TRPM4	ARHGEF25	-0.338	7.00146376309801e-15
TRPM4	AC104785.1	0.338	7.16049028560228e-15
TRPM4	MEIS3P1	-0.336	1.07600110666213e-14
TRPM4	SOX7	-0.335	1.13533892366326e-14
TRPM4	MGST1	0.334	1.46358510852681e-14
TRPM4	COL21A1	-0.334	1.55070890385371e-14
TRPM4	NLRP1	-0.334	1.64773224148648e-14
TRPM4	GJC1	-0.334	1.65309550201481e-14
TRPM4	RIPK3	-0.333	1.88497537363784e-14
TRPM4	ZBTB47	-0.332	2.05979712790367e-14
TRPM4	KIAA1671	-0.332	2.10924746257837e-14
TRPM4	FYN	-0.332	2.42668543574794e-14

TRPM4	KBTBD6	-0.331	2.89265485321288e-14
TRPM4	SIN3B	-0.331	2.92448401219386e-14
TRPM4	HVCN1	-0.330	3.25142816055993e-14
TRPM4	SV2A	-0.330	3.54152815953571e-14
TRPM4	DGLUCY	-0.329	3.66890750115346e-14
TRPM4	DYNC2I1	-0.329	3.77899332993009e-14
TRPM4	NFASC	-0.329	3.82531036428282e-14
TRPM4	ST6GALNAC2	-0.328	4.52672326939386e-14
TRPM4	PAK5	-0.328	4.7322569328767e-14
TRPM4	SLC25A43	-0.327	5.51773610365829e-14
TRPM4	TMCC2	-0.327	6.07365854531977e-14
TRPM4	CCN4	-0.326	6.71580598871778e-14
TRPM4	NRG1	-0.325	7.98739509698734e-14
TRPM4	MAP3K8	-0.325	8.13517132390809e-14
TRPM4	DUOX1	-0.325	8.21140896591039e-14
TRPM4	ACSS3	-0.324	9.32278397422036e-14
TRPM4	NUDT9P1	-0.324	9.77818778322444e-14
TRPM4	ANXA6	-0.324	1.0533151103882e-13
TRPM4	KDM8	0.323	1.08365447085084e-13
TRPM4	KCNQ3	-0.323	1.12182148379564e-13
TRPM4	SPATA6	-0.323	1.14563029088637e-13
TRPM4	PPP1R18	-0.323	1.15892655472334e-13
TRPM4	LINC01679	-0.323	1.17776557460566e-13
TRPM4	AL133482.1	-0.323	1.22052899315784e-13
TRPM4	PNMA8A	-0.323	1.2277867149461e-13
TRPM4	SRSF4	-0.323	1.23124527509304e-13
TRPM4	HARS2	-0.323	1.24916833314585e-13
TRPM4	FAM110C	-0.322	1.31864956774692e-13
TRPM4	TMEM139	-0.322	1.43916138517744e-13
TRPM4	FGD5	-0.322	1.4990336764233e-13
TRPM4	AC111152.2	0.322	1.54849033658731e-13
TRPM4	JAM3	-0.321	1.63387229492546e-13
TRPM4	LINC01963	-0.321	1.69690120126797e-13
TRPM4	CCDC8	-0.321	1.82950325246703e-13
TRPM4	CAMK2G	-0.321	1.8431926968297e-13
TRPM4	ITGB4	-0.320	2.01967500922453e-13
TRPM4	SEPTIN8	-0.320	2.05965390812428e-13
TRPM4	LAMA3	-0.320	2.18537967756096e-13
TRPM4	COL8A2	-0.320	2.2287516781449e-13
TRPM4	TMX3	-0.319	2.63745494307682e-13
TRPM4	USP11	-0.318	2.71742132075931e-13
TRPM4	CASP1	-0.318	2.77036476609961e-13
TRPM4	RPL13AP7	0.318	2.79918622415123e-13
TRPM4	SLC12A4	-0.318	2.85997489957154e-13

TRPM4	AKAP8	-0.318	3.10156020079174e-13
TRPM4	CALHM2	-0.317	3.59532956894372e-13
TRPM4	STX2	-0.316	4.04359741763465e-13
TRPM4	AL354861.3	-0.316	4.11721486603606e-13
TRPM4	KBTBD7	-0.316	4.13629701221614e-13
TRPM4	PPP1R37	0.316	4.39834006566315e-13
TRPM4	RRN3P1	-0.315	4.79443607448868e-13
TRPM4	MEIS3P2	-0.314	5.64801463390755e-13
TRPM4	GGA2	-0.314	5.84846684183839e-13
TRPM4	ST6GALNAC4	-0.314	5.88511816880003e-13
TRPM4	TBC1D2	-0.314	5.93429092894431e-13
TRPM4	ZNF488	-0.314	6.17891395772187e-13
TRPM4	ANKRD44	-0.314	6.45912183290008e-13
TRPM4	PICART1	-0.313	6.76228592505375e-13
TRPM4	CCDC82	-0.313	7.62077171168713e-13
TRPM4	C1S	-0.312	8.13662058519402e-13
TRPM4	PLD1	-0.312	9.02425787912767e-13
TRPM4	CDHR1	-0.311	1.00769245264248e-12
TRPM4	CABLES1	-0.311	1.01945061464567e-12
TRPM4	AC092953.2	-0.311	1.07576233112221e-12
TRPM4	WDR19	-0.310	1.18915615929065e-12
TRPM4	RADX	-0.310	1.21510630086277e-12
TRPM4	LRP4	-0.309	1.36508902073468e-12
TRPM4	ARHGAP25	-0.309	1.43330742034175e-12
TRPM4	PER2	-0.309	1.49872092697403e-12
TRPM4	AC107983.1	0.308	1.6515190152256e-12
TRPM4	AC024293.1	0.308	1.71167097180256e-12
TRPM4	COL27A1	-0.307	1.89079826288093e-12
TRPM4	DOK4	-0.307	2.00981775154461e-12
TRPM4	DUOXA1	-0.307	2.13828413776639e-12
TRPM4	IFFO2	-0.306	2.27330899112906e-12
TRPM4	STING1	-0.306	2.40738711163408e-12
TRPM4	FILIP1L	-0.306	2.44149237168563e-12
TRPM4	NMUR1	-0.306	2.57799160100436e-12
TRPM4	TNFAIP3	-0.306	2.59657888417483e-12
TRPM4	IRF8	-0.306	2.6257544088875e-12
TRPM4	AL590434.1	-0.306	2.63004948830805e-12
TRPM4	VSNL1	-0.306	2.63551782180032e-12
TRPM4	NEDD9	-0.305	2.69175230410568e-12
TRPM4	GJD3	-0.305	2.76940544274531e-12
TRPM4	ASB1	-0.305	2.9100065231444e-12
TRPM4	TRIM29	-0.305	2.9281049864192e-12
TRPM4	SLC3A2	0.304	3.16101911312819e-12
TRPM4	AL445524.1	0.304	3.3274314224685e-12

TRPM4	TSHZ3	-0.304	3.38521340423125e-12
TRPM4	KIF3C	-0.303	3.88993062457097e-12
TRPM4	STARD9	-0.303	4.10614606555079e-12
TRPM4	ATG101	0.303	4.11673390324446e-12
TRPM4	ARMCX4	-0.303	4.12632058492493e-12
TRPM4	CAMK4	-0.303	4.37414823085104e-12
TRPM4	TRPV4	-0.302	4.45750903708057e-12
TRPM4	SGCE	-0.302	4.87716211448966e-12
TRPM4	VPS9D1-AS1	0.302	4.90931172337474e-12
TRPM4	PRELID1P6	0.301	5.35648257900455e-12
TRPM4	AC090559.1	-0.301	6.00078218527129e-12
TRPM4	MPEG1	-0.300	6.40769214667466e-12
